# Pressure‐Activated and Retained Blue Emission in Melamine Through Hydrogen‐Bond and Packing Regulation

**DOI:** 10.1002/advs.77871

**Published:** 2026-09-27

**Authors:** Siyao Wang, Weiwei Dong, Shuo Yang, Jiayue Jiang, Yushuang Wang, Shuailing Ma, Peng Liu, Xiaodong Li, Haitao Wang

**Affiliations:** ^1^ School of Materials Science and Engineering Jilin University Changchun China; ^2^ High Energy Photon Source, Institute of High Energy Physics Chinese Academy of Sciences Beijing China; ^3^ School of Physical Science and Technology & Institute of High‐Pressure Physics Ningbo University Ningbo China

**Keywords:** high pressure, hydrogen bonding, melamine, organic luminescence, pressure engineering

## Abstract

Pressure provides a direct way to regulate molecular packing and excited‐state relaxation in organic crystals, but retaining pressure‐induced emission after decompression remains challenging. Here, we report pressure‐activated and retained blue emission in melamine (MA), a simple nitrogen‐rich molecular crystal assembled by N–H···N hydrogen bonds. MA shows weak blue emission at ambient conditions, whereas compression markedly enhances its photoluminescence and pressure release produces an even brighter state, with an approximately 35‐fold increase in integrated emission intensity. Time‐resolved photoluminescence and UV–vis absorption measurements indicate that the retained enhancement is not mainly governed by lifetime prolongation or optical‐absorption changes. In situ Raman, FTIR, and powder X‐ray diffraction reveal pressure‐induced changes in molecular and lattice vibrations, the N─H‐related hydrogen‐bonding environment, and anisotropic lattice compression, together with increasing structural disorder at higher pressures and incomplete recovery after decompression. TD‐DFT calculations show that N─H···N shortening slightly increases the S_1_ oscillator strength and modifies the local hole–electron distribution, qualitatively supporting the influence of hydrogen‐bond modulation on the excited‐state properties. Together, these results associate the retained emission with an incompletely recovered pressure‐modified intermolecular and lattice environment, highlighting hydrogen‐bonded molecular crystals for pressure‐activated and retained luminescence.

## Graphical abstract

Pressure treatment activates and retains bright blue emission in melamine (MA). The pressure‐modified hydrogen‐bonding network and molecular packing create a distinct local environment after decompression, changing the balance among competing excited‐state relaxation pathways and enabling retained emission.

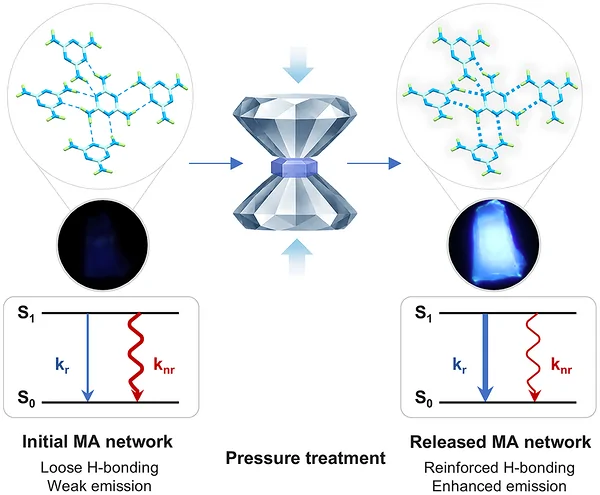

## 1 Introduction

Stimulus‐responsive organic luminescent materials have attracted increasing attention because of their potential applications in information encryption, optical data storage, anti‐counterfeiting, sensing, and pressure visualization [1, 2, 3]. Among various external stimuli, pressure is particularly powerful because it can precisely tune intermolecular distances, molecular packing, and weak noncovalent interactions without changing chemical composition [4, 5, 6, 7, 8, 9, 10]. By regulating lattice distortion, intermolecular interactions, and excited‐state relaxation, pressure has enabled emission enhancement, color modulation, and even retention of pressure‐generated emissive states across organic crystals and hybrid luminescent materials [11, 12, 13, 14]. These structural changes can directly affect excited‐state relaxation pathways in organic molecular crystals. In many cases, compression enhances intermolecular coupling and activates nonradiative decay, resulting in emission quenching or pressure‐induced spectral shifts [15, 16, 17, 18]. Pressure‐induced emission enhancement (PIEE), by contrast, reflects a different balance between radiative and nonradiative processes, usually involving restricted molecular motion, lattice rigidification, or favorable intermolecular interactions [19, 20, 21, 22, 23, 24]. A further challenge is to retain the pressure‐induced emissive state after pressure release, because most pressure‐responsive organic systems recover their initial optical properties once the external pressure is removed. Such reversibility limits the use of pressure as a practical way to preserve optimized emissive states. Retained emission after decompression usually requires partial preservation of the pressure‐modified local structure or intermolecular environment at ambient conditions. Recent studies have demonstrated retained luminescence after pressure treatment through hydrogen‐bond strengthening or reconfiguration, π‐stacking rearrangement, intermolecular charge‐transfer regulation, and coordination‐environment reconstruction, as exemplified by TPA, IPA, INA, BA, and Zn‐IPA [25, 26, 27, 28, 29]. Therefore, molecular systems that combine pressure‐activated emission with retained emission are particularly useful for clarifying how pressure‐treated packing structures regulate organic luminescence.

Hydrogen‐bonded nitrogen‐rich molecular crystals are especially suitable models for studying this issue. Their abundant N─H donors and heteroatom acceptors generate extended N─H···N or N─H···X networks, in which hydrogen‐bond geometry, packing density, and molecular vibrations can respond strongly to compression [30, 31, 32, 33, 34, 35]. These changes may alter local rigidity and the balance between radiative and nonradiative decay. For amino‐functionalized small molecules, such regulation is expected to restrict vibration‐ or rotation‐assisted energy loss while simultaneously modifying the electronic distribution of excited states. However, how hydrogen‐bonded packing contributes to pressure‐activated and retained emission remains insufficiently understood, especially in simple molecular crystals without deliberately designed fluorophores.

In this work, we selected melamine (MA), a commercially available amino‐functionalized s‐triazine crystal, as a simple hydrogen‐bonded model system [36, 37, 38, 39, 40, 41]. MA has a well‐defined hydrogen‐bonded crystal structure but does not contain an extended π‐conjugated fluorophore, making it suitable for examining how local packing and intermolecular interactions regulate emission under pressure. In situ high‐pressure photoluminescence (PL) measurements show that MA exhibits pressure‐activated blue emission and retains a much brighter emissive state after pressure release, with the integrated PL intensity remaining approximately 35 times higher than that of the initial sample after decompression. To understand the origin of this behavior, we further combined in situ time‐resolved PL, UV–vis absorption, Raman and FTIR spectroscopy, synchrotron powder X‐ray diffraction (XRD), Hirshfeld surface analysis, and TD‐DFT calculations. These analyses indicate that this retained enhancement is not primarily governed by lifetime prolongation or optical‐absorption changes, but is associated with incomplete recovery of the pressure‐reorganized hydrogen‐bonded packing and vibrational environment, leaving a metastable local structural state after decompression. This work provides a simple molecular platform for understanding retained pressure‐enhanced luminescence in hydrogen‐bonded organic crystals.

## 2 Results and Discussion

### 2.1 Molecular Structure and Pressure‐Activated Emission

**FIGURE 1:**
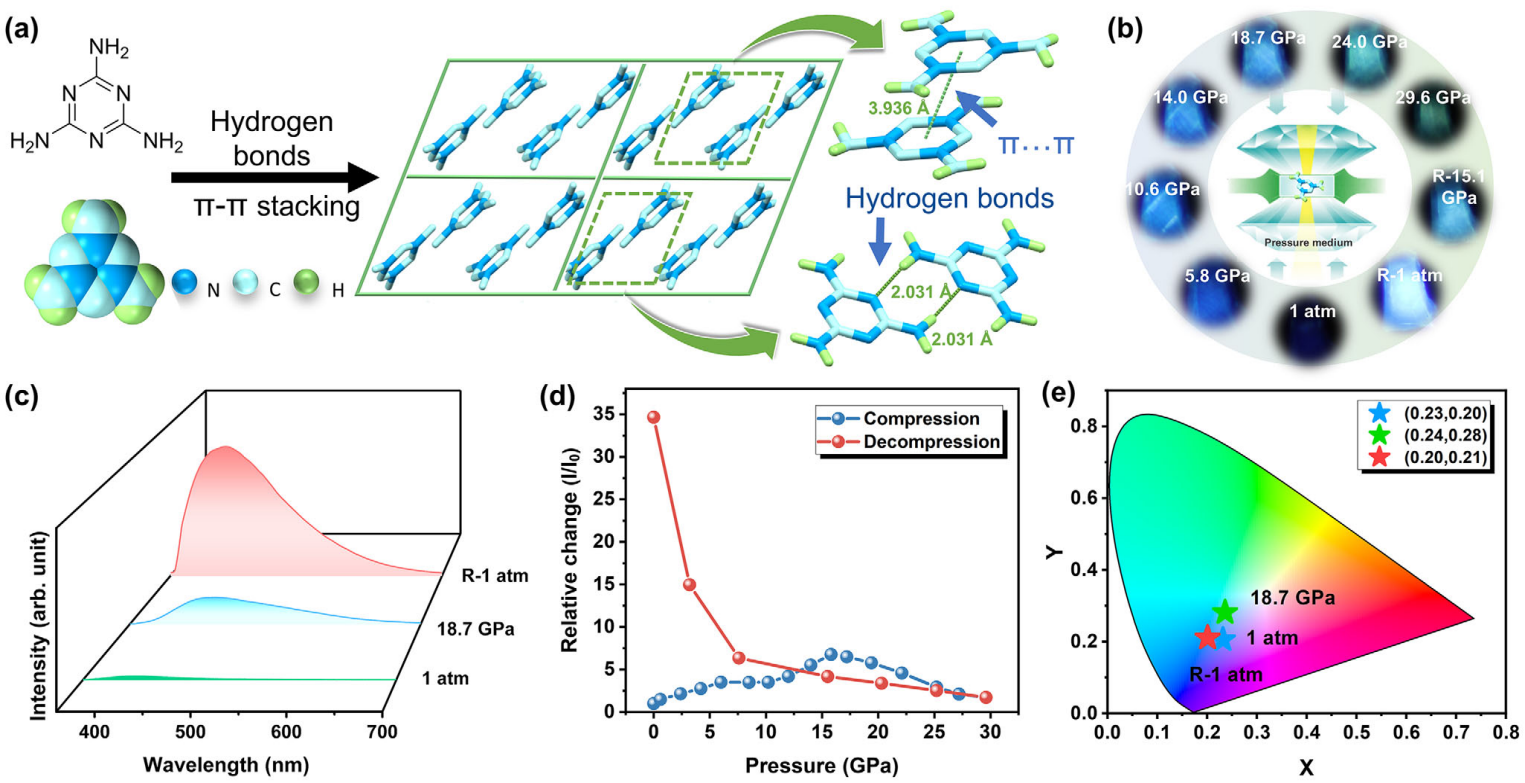
Crystal structure and pressure‐regulated emission behavior of MA. (a) Molecular structure, frontier orbital distribution, and crystal packing of MA, highlighting the hydrogen‐bonded molecular layer and weak interlayer contacts. (b) Optical photographs under UV excitation during compression and after pressure release. (c) PL spectra at 1 atm, 18.7 GPa and after complete pressure release. (d) Relative integrated PL intensity during compression and decompression. (e) CIE chromaticity coordinates at selected pressure states.

MA is a nitrogen‐rich molecular crystal composed of a nearly planar s‐triazine core and three peripheral amino groups. At ambient conditions, MA crystallizes in the monoclinic P2_1_/a space group. Le Bail profile fitting of our ambient‐pressure laboratory Cu Kα PXRD pattern gives lattice parameters of a = 10.5956(20) Å, b = 7.47635(47) Å, c = 7.2808(12) Å, β = 112.1635(32)°, and V = 533.992(81) Å^3^, which are in close agreement with the reference crystal structure [42]. As shown in Figure 1a, adjacent MA molecules are connected through abundant N─H···N hydrogen bonds to form ordered molecular layers, while the interlayer packing is further stabilized by weak close contacts and π‐related interactions. This supramolecular packing is dominated by compressible noncovalent interactions and provides the structural basis for the pressure response of MA. The UV–vis absorption spectrum of ambient MA is mainly located in the ultraviolet region, consistent with excitation of the s‐triazine framework (Figure S1). Under UV excitation, the crystal exhibits only weak blue emission, indicating that efficient nonradiative energy loss pathways remain active in the initial packing environment.

We therefore performed in situ high‐pressure PL measurements in a diamond anvil cell. The optical photographs in Figure 1b show that the initially weak blue emission becomes much brighter upon compression, accompanied by a slight color change from blue toward greenish blue/cyan. The sample remains visibly emissive at higher pressure. Notably, after complete pressure release, the recovered sample displays a much brighter blue emission than the original crystal, demonstrating that the pressure‐induced emission enhancement is partially retained at ambient conditions. The corresponding PL spectra and integrated intensity plot confirm this visual observation (Figure 1c,d). During compression, the emission intensity increases markedly and reaches a maximum at 18.7 GPa, followed by a moderate decrease in intensity at higher pressures, while the intensity remains much stronger than that of the initial sample. After complete pressure release, the integrated PL intensity is approximately 35 times higher relative to the initial state (Figure 1d).

**FIGURE 2:**
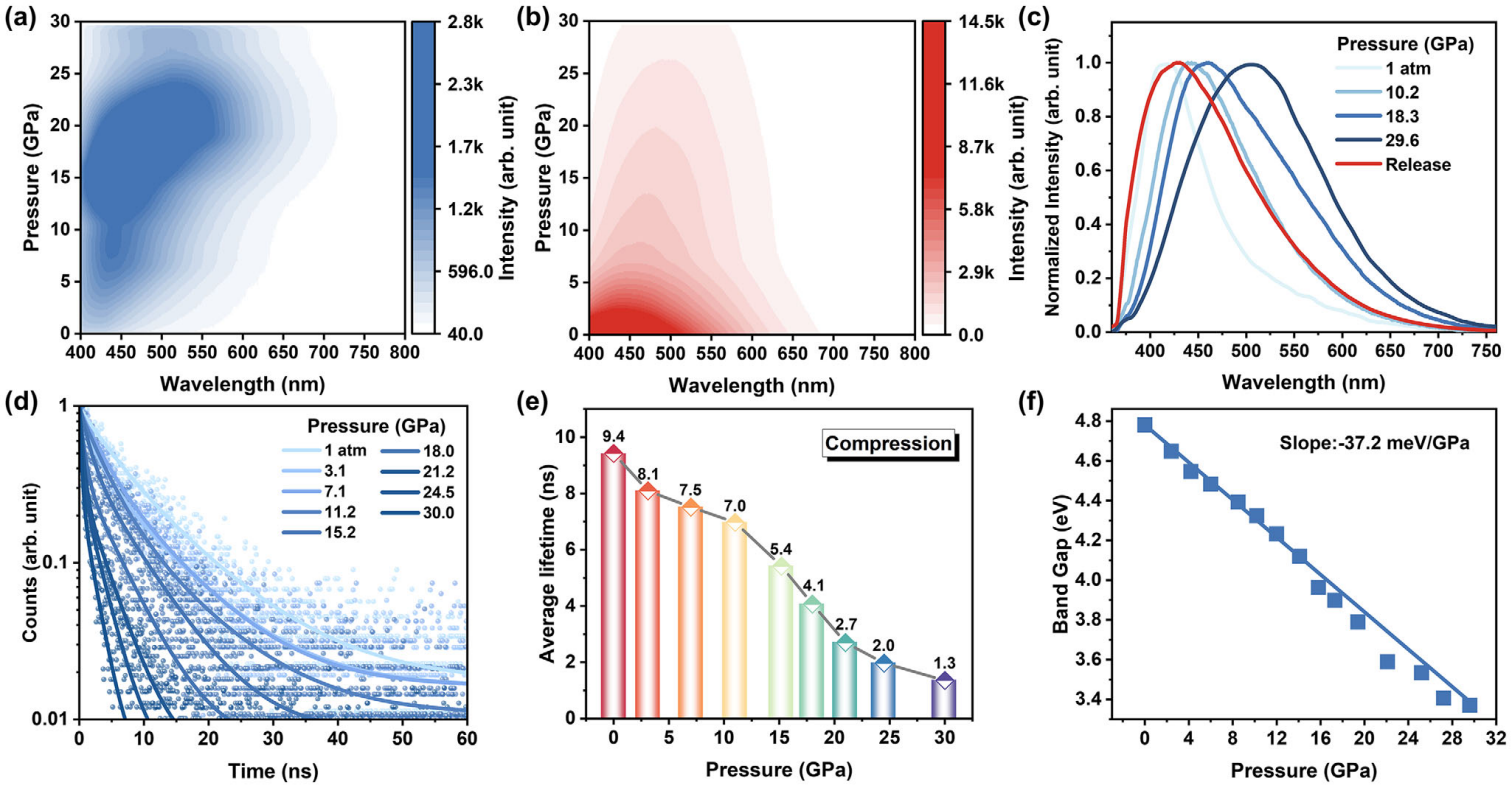
Pressure‐dependent photophysical evolution of MA. (a) PL intensity map during compression. (b) PL intensity map during decompression. (c) Normalized PL spectra at selected pressure states. (d) Time‐resolved PL decay curves during compression. (e) Average lifetime evolution during compression. (f) Pressure‐dependent optical band‐gap evolution during compression.

The pressure‐dependent PL maps give a more complete view of this nonmonotonic emission evolution: during compression, the emission signal first strengthens and then weakens, whereas strong emission is recovered during decompression (Figure 2a,b). In parallel, the PL spectra, normalized PL spectra and CIE coordinates show a slight redshift during compression and move back toward the blue region after decompression (Figures 1c,e and 2c). No new dominant long‐wavelength emission band appears during the whole pressure cycle, indicating that pressure mainly regulates the local environment of the original emissive state rather than generating a new emissive center.

We further performed time‐resolved PL measurements to examine the excited‐state decay dynamics under pressure. As shown in Figure 2d,e, the PL decay becomes faster with increasing pressure, and the average lifetime decreases from 9.4 ns at ambient pressure to 1.3 ns at 30.0 GPa. This monotonic lifetime shortening contrasts with the nonmonotonic PL intensity evolution, showing that the pressure‐enhanced emission is not driven by a longer‐lived excited state. The PL lifetime reflects the overall rate of excited‐state depopulation, whereas the steady‐state PL intensity depends on the fraction of excitations that decay radiatively. Therefore, the stronger PL observed at moderate pressure together with a shorter lifetime indicates that compression changes the relative contributions of the competing excited‐state relaxation pathways. The contrasting trends therefore indicate a pressure‐dependent redistribution of excited‐state relaxation pathways rather than a simple monotonic change in a single decay channel. During decompression, the decay profiles remain in the nanosecond range and change only moderately (Figure S4), confirming the fluorescence‐like nature of the emission and further indicating that the retained bright state is not governed by lifetime prolongation.

We also examined the pressure‐dependent UV–vis absorption spectra to evaluate the contribution of optical absorption to the retained emission. Upon compression, the absorption edge gradually shifted to longer wavelengths, and the optical bandgap decreased nearly linearly with a slope of −37.2 meV GPa^−1^ (Figure 2f and Figure S3), reflecting pressure‐induced modulation of the electronic environment. During decompression, however, the spectral changes in absorption were much smaller than the pronounced enhancement in PL intensity, indicating that the retained bright emission cannot be explained by optical absorption alone. Since the PL spectra show no new dominant emission band and the lifetime does not increase after pressure treatment, the retained emission is more likely related to changes in the local molecular environment that affect nonradiative relaxation.

**FIGURE 3:**
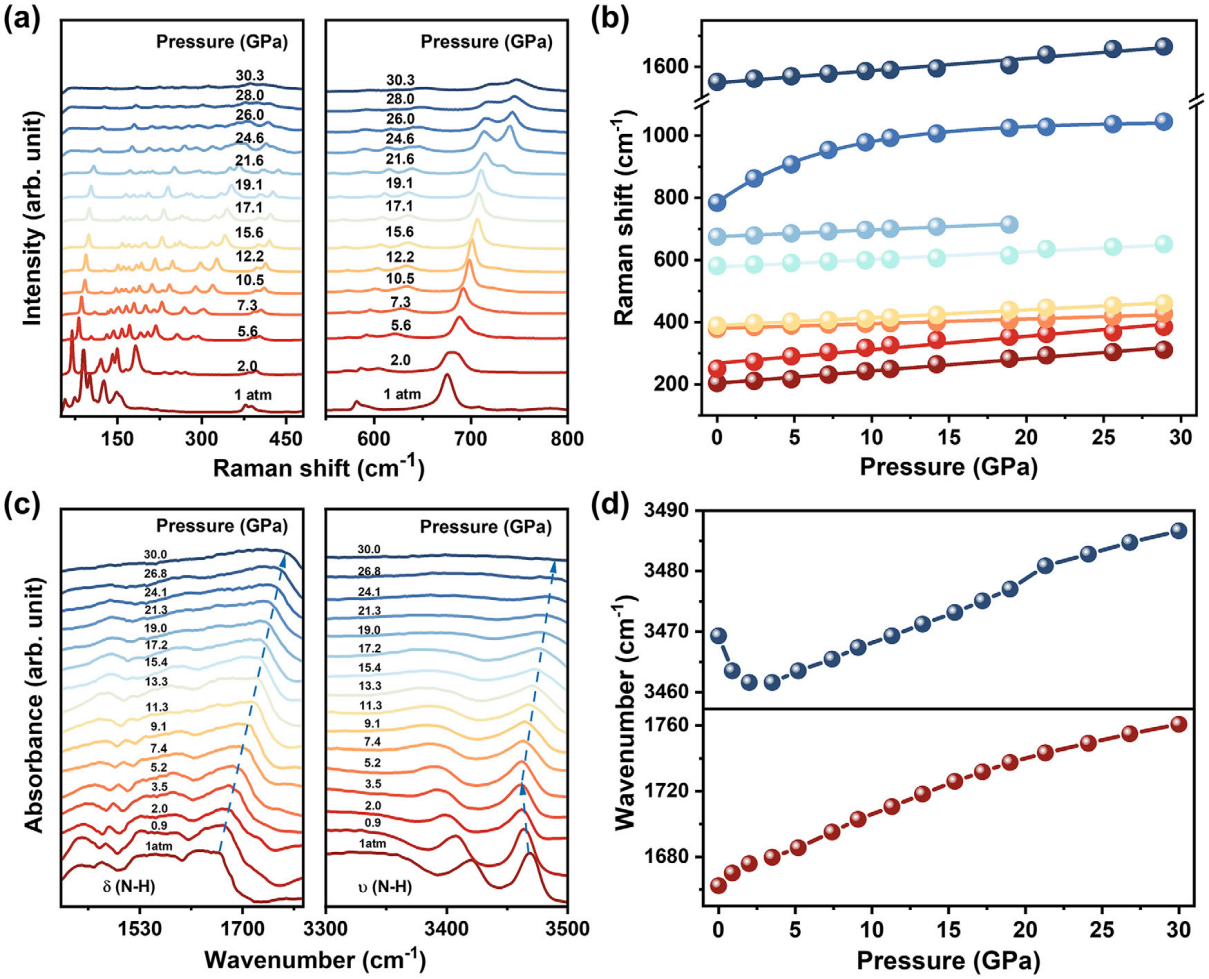
In situ Raman and FTIR characterization of pressure‐induced vibrational evolution in MA. (a) Selected in situ high‐pressure Raman spectra. (b) Pressure‐dependent Raman shifts of representative vibrational modes. (c) Selected in situ high‐pressure FTIR spectra in the N─H bending and N─H stretching regions. (d) Pressure‐dependent wavenumber evolution of the N─H stretching vibration, ν(N─H), and N─H bending vibration, δ(N─H).

We therefore turned to Raman and FTIR spectroscopy to examine how pressure modifies the molecular vibrations and N─H‐related hydrogen‐bonding environment of MA. As shown in Figure 3a,b, the Raman spectra of MA exhibit pressure‐dependent frequency shifts, peak broadening, and pronounced relative‐intensity changes during compression. The low‐wavenumber modes are mainly related to lattice vibrations and associated with intermolecular motions, while the higher‐wavenumber modes can be assigned to triazine‐ring skeletal and amino‐group‐related vibrations. During decompression, the Raman spectra do not fully recover to the initial profile (Figures S6 and S7), particularly in the low‐frequency region, indicating incomplete recovery of the pressure‐modulated vibrational environment.

To further clarify the structural response of MA in the low‐frequency region, we analyzed the Raman spectra during the initial compression stage in detail (Figures S8 and S9). The most pronounced spectral change occurs from 1 atm to 1.1 GPa, with substantial redistribution of relative intensities and changes in peak positions and spectral profiles. Periodic lattice‐dynamics calculations of monoclinic melamine indicate that this region is dominated by closely spaced external lattice modes involving collective molecular translations, librations, and mixed translational–rotational motions, making them particularly sensitive to intermolecular contacts and molecular packing [43]. Given the intermolecular character of these low‐frequency modes, this initial response indicates a rapid adjustment of the soft intermolecular degrees of freedom and the associated packing environment. Above 1.1 GPa, the reliably tracked low‐frequency modes evolve more regularly toward higher wavenumbers, indicating progressive lattice stiffening as intermolecular distances decrease and molecular motions become increasingly constrained. Thus, the low‐frequency Raman results reveal an initial rapid adjustment of the intermolecular environment and lattice dynamics, followed by progressive stiffening of the molecular lattice upon further compression.

FTIR spectra further reveal the pressure‐induced modulation of the N─H‐related environment. In the 750–1800 cm^−1^ region, bands associated with coupled N─H bending and triazine‐ring C─N/C═N skeletal vibrations shift progressively during compression (Figure S10). In the N─H stretching and bending regions (Figure 3c,d), δ(N─H) exhibits a continuous blueshift, indicating restricted amino‐group bending motion, whereas ν(N─H) vibration shows a slight redshift at the initial stage followed by a blueshift at higher pressure, which should be considered as a collective response of the hydrogen‐bonded network under compression rather than a simple monotonic change of an isolated N─H bond. After pressure release, the FTIR spectra remain different from those of the initial MA, especially in the N─H stretching and bending regions (Figures S10 and S11). Together with the Raman results, this incomplete recovery indicates that pressure treatment partially retains a modified local vibrational and N─H‐related environment, which is consistent with the retained emission enhancement.

**FIGURE 4:**
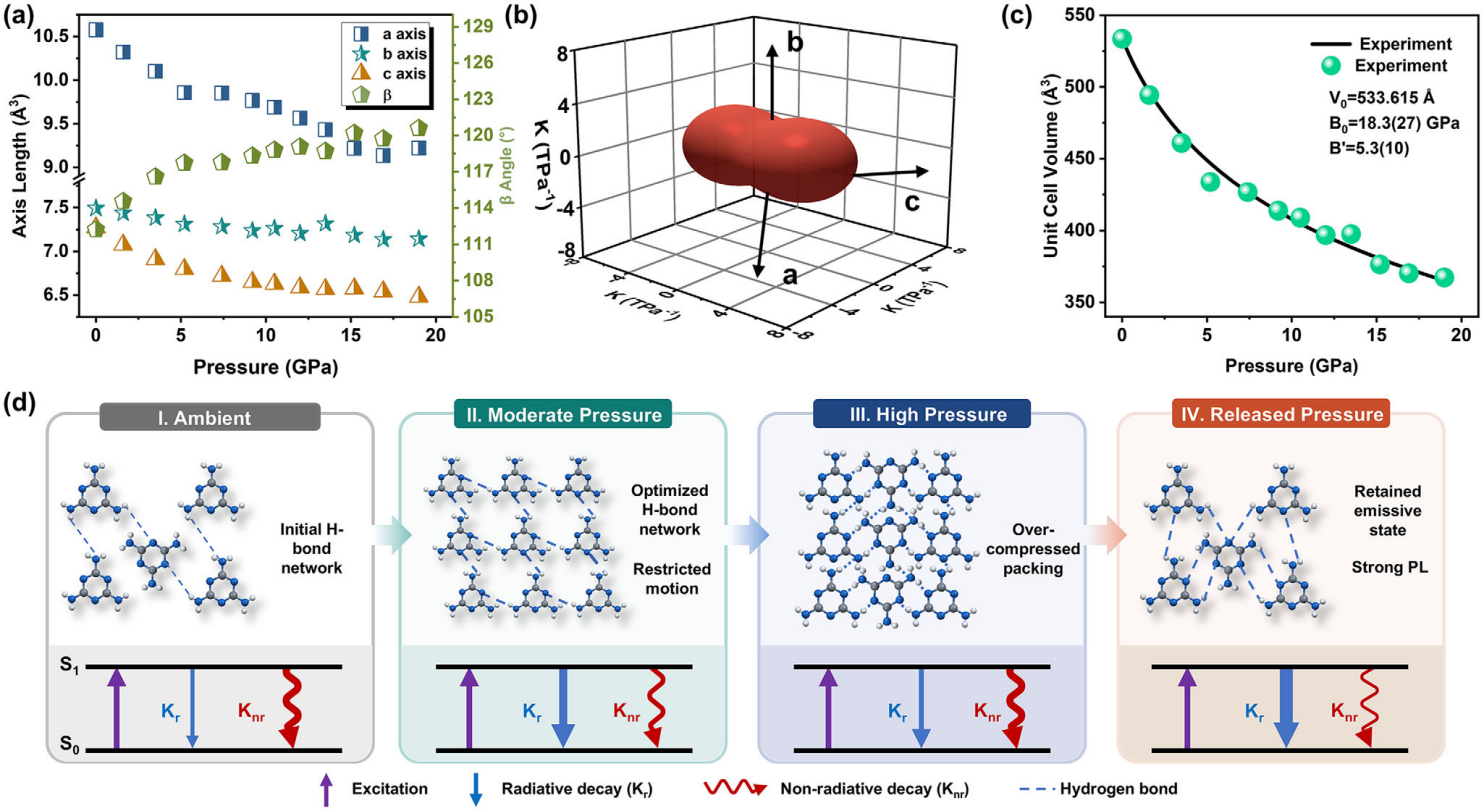
Pressure‐modulated lattice response and proposed emission mechanism of MA. (a) Pressure‐dependent evolution of lattice parameters and β angle. (b) Three‐dimensional compressibility indicatrix showing the anisotropic lattice compression of MA. (c) Pressure‐dependent unit‐cell volume fitted using the third‐order Birch–Murnaghan equation of state. (d) Schematic illustration of the pressure‐dependent evolution of the hydrogen‐bonded packing and the corresponding radiative and nonradiative decay pathways in the initial, moderately compressed, highly compressed, and pressure‐released states.

In situ high‐pressure XRD shows that the diffraction peaks of MA progressively shift toward higher angles and become broader and weaker upon compression, reflecting continuous lattice contraction accompanied by a gradual reduction in long‐range structural order (Figure S12). Because of the pronounced peak broadening and overlap at intermediate and high pressures, quantitative unit‐cell analysis was restricted to below 20 GPa. Within this pressure range, Le Bail profile fitting reveals pronounced anisotropic lattice compression (Figure 4a,b, Figure S13, and Table S1). A third‐order Birch–Murnaghan fit to the corresponding pressure–volume data gives B_0_ = 18.3(27) GPa and B’ = 5.3(10) (Figure 4c). At higher pressures, the continued broadening and weakening of the Bragg reflections indicate a further reduction in long‐range lattice order and the development of pronounced structural disorder, with partial amorphization developing at higher pressures.

This pressure‐induced structural evolution correlates with the enhancement–quenching behavior of the PL. At low and moderate pressures, anisotropic lattice contraction produces a more compact intermolecular packing environment and enhances the local constraints associated with N─H···N interactions, thereby restricting molecular and lattice motions and favoring emission enhancement. Upon further compression beyond the PL maximum at 18.7 GPa, increasing lattice strain and structural disorder progressively perturb the ordered intermolecular packing, counteracting the beneficial rigidification established at lower pressures and leading to a decrease in PL intensity. The nonmonotonic emission response of MA therefore reflects a competition between favorable intermolecular rigidification at moderate pressure and structural perturbation induced by overcompression at higher pressure.

After complete pressure release, MA does not return to its initial crystalline state. The recovered sample retains the major diffraction features associated with the pristine structure, but the reflections are substantially broadened and weakened, with partial merging of several diffraction features (Figure S14a). Line‐shape analysis of three representative diffraction components, Q1–Q3, under identical fitting conditions shows FWHM increases of 47.5%, 71.1%, and 45.2%, respectively, relative to the initial state (Figures S15 and S16), indicating that the pressure‐induced structural perturbation is not fully relaxed after decompression. Together with the incomplete recovery of the Raman and FTIR spectra, these results suggest that the recovered MA retains its crystalline framework while its lattice order and local intermolecular environment remain distinct from those of the pristine crystal. During decompression, the severe strain and unfavorable structural perturbations associated with strong compression are released, whereas part of the pressure‐modified N─H‐related local environment and short‐range packing is retained. The resulting pressure‐treated state is therefore distinct from both the pristine crystal and the in situ highly compressed state, and this coexistence of structural relaxation and local structural retention is consistent with the renewed and persistent enhancement of the blue emission after pressure release.

**FIGURE 5:**
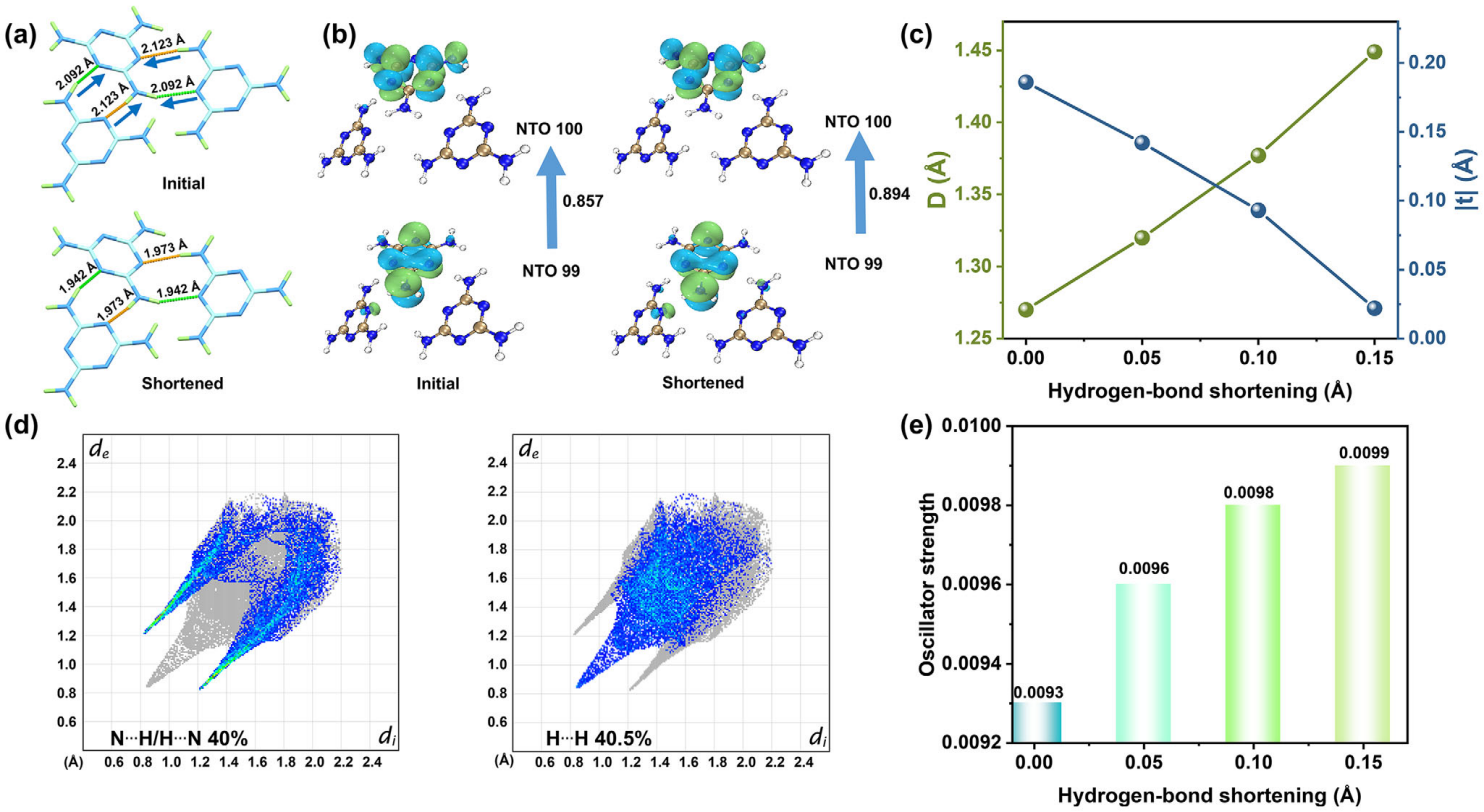
Theoretical insight into hydrogen‐bond‐regulated excited‐state properties of MA. (a) Hydrogen‐bond‐shortening model extracted from the MA crystal. (b) Representative S_1_ natural transition orbitals (NTOs) before and after hydrogen‐bond shortening. (c) Evolution of the hole–electron separation index D and |t| value as a function of hydrogen‐bond shortening. (d) Two‐dimensional fingerprint plots showing N···H/H···N and H···H intermolecular contacts. (e) Evolution of the S_1_ oscillator strength as a function of hydrogen‐bond shortening.

Having established the experimental correlation between retained emission and pressure‐modulated hydrogen‐bonded packing, we next used theoretical calculations to examine how changes in the N─H···N hydrogen‐bonding environment affect the excited‐state transition properties of MA. Based on the Raman, FTIR, and XRD results, a hydrogen‐bond‐shortening model was constructed to qualitatively simulate local hydrogen‐bond compression under pressure. As illustrated in Figure 5a, a hydrogen‐bonded aggregate was extracted from the MA crystal, and selected N─H···N contacts were shortened by 0.00, 0.05, 0.10, and 0.15 Å along the hydrogen‐bonding direction. This simplified model was used to evaluate the qualitative influence of local hydrogen‐bond compression on the excited‐state properties, rather than any specific pressure state.

Hirshfeld surface and two‐dimensional fingerprint analyses support the structural relevance of this model [44]. N···H/H···N contacts contribute approximately 40.0% of the intermolecular contacts, while H···H contacts account for approximately 40.5% (Figure 5d), confirming that hydrogen‐bonded contacts play a major role in the MA packing network. Comparative calculations on monomer, π‐stacked dimer, and hydrogen‐bonded aggregate models further highlight the role of local packing (Figure S17 and Table S2). Within these simplified models, the π‐stacked dimer exhibits an almost negligible S_1_ oscillator strength, whereas the hydrogen‐bonded aggregate shows a relatively higher value, suggesting that the N─H···N environment can influence the electronic transition probability. Reference hole, electron, and transition‐density distributions are shown in Figure S18.

NTO analysis shows that the S_1_ state of the hydrogen‐bonded aggregate is dominated by the NTO99 → NTO100 transition (Figure 5b, and Figure S19) [45]. With progressive hydrogen‐bond shortening, the contribution of this dominant transition increases from 85.7% to 89.4%, accompanied by a slight increase in the S_1_ oscillator strength from 0.0093 to 0.0099 (Figure 5e, Figure S20, and Table S2). Hole–electron analysis using Multiwfn further shows that the D value increases from 1.270 to 1.449 Å, whereas |t| decreases from 0.186 to 0.022 Å (Figure 5c, Figure S21, and Table S3), indicating a locally changed excited‐state distribution rather than a strong long‐range charge‐transfer state [46, 47, 48, 49]. Although this increase in oscillator strength is modest and cannot quantitatively account for the approximately 35‐fold experimental PL enhancement by itself, it qualitatively supports the idea that local N─H···N hydrogen‐bond compression can contribute to an increased probability of radiative transition. The much larger experimental enhancement likely arises from the combined effects of this electronic‐structure modulation and pressure‐induced changes in molecular packing and lattice vibrations. The resulting restriction of molecular and lattice motions and modification of the vibrational environment can alter the competing excited‐state relaxation pathways, thereby contributing to the pressure‐activated emission. Partial retention of the pressure‐modified packing and vibrational environment after decompression may further account for the retained emission enhancement.

In the broader context of retained pressure‐responsive luminescence, representative pressure‐treated materials are compared in Table S4, highlighting the distinctive simplicity of MA as a commercially available molecular crystal lacking an extended π‐conjugated fluorophore. Related studies have shown that pressure treatment can generate persistent or switchable emissive states in organic molecular materials, providing opportunities for post‐treatment optical functions [25, 26, 27, 28, 29]. Additional measurements summarized in Figure S2 further demonstrate the reproducibility and post‐treatment evolution of the pressure‐released emissive state of MA. Three independently loaded samples reproduce the characteristic enhancement–attenuation–retention behavior, with released‐state PL enhancement factors of approximately 35, 35, and 38 relative to their corresponding initial states. After decompression, the retained emission gradually decreases and redshifts during a seven‐day observation period, but remains approximately twice as intense as that of pristine MA after seven days. This correlated intensity decay and spectral evolution are consistent with slow relaxation of the pressure‐generated metastable local environment. Notably, heating the recovered sample to 55°C and 100°C followed by cooling to room temperature does not restore the initial weak‐emission state, indicating that the retained emissive state is not readily erased by moderate thermal treatment. Together, these results show that pressure treatment produces a reproducible and metastable optical state that persists after removal of the external pressure and remains distinguishable from pristine MA over the monitored period. The retained optical contrast between pristine and pressure‐treated MA suggests potential applications in pressure‐responsive optical encoding, temporary information storage, and anti‐counterfeiting, while further spatial pressure patterning and scalable processing will be required to evaluate such applications.

## 3 Conclusions

In summary, we demonstrate pressure‐activated and retained blue emission in melamine. Upon compression, the integrated PL intensity first increases and reaches a maximum at 18.7 GPa, followed by a moderate decrease at higher pressures. After complete pressure release, the recovered sample retains bright blue emission, with an approximately 35‐fold increase in integrated PL intensity compared with the initial MA sample. Time‐resolved PL and UV–vis absorption measurements show that this retained bright emission is not mainly governed by lifetime prolongation or optical‐absorption changes. Raman, FTIR, and powder XRD analyses reveal pressure‐induced modulation of molecular vibrations and N─H‐related local environments, anisotropic lattice contraction at lower pressures, increasing structural disorder at higher pressures, and incomplete structural recovery after pressure release. TD‐DFT calculations further suggest that compression of the N─H···N hydrogen‐bonding environment increases the S_1_ oscillator strength and modifies the local hole–electron distribution, qualitatively supporting its influence on the excited‐state electronic properties. Taken together, these observations associate the pressure‐activated and retained emission of MA with a pressure‐treated metastable local structural state characterized by incomplete recovery of the reorganized hydrogen‐bonding environment and residual structural disorder. This modified local environment is consistent with a changed balance among competing excited‐state relaxation pathways, allowing the bright‐blue emission to be retained after decompression.

## Author Contributions

**Siyao Wang**: methodology, data curation, formal analysis, validation, investigation, visualization, writing – original draft. **Weiwei Dong**: conceptualization, methodology, supervision, validation, funding acquisition, project administration, resources, writing – review and editing. **Shuo Yang**: writing – review and editing, data curation, investigation. **Jiayue Jiang**: writing – review and editing. **Yushuang Wang**: writing – review and editing. **Shuailing Ma**: writing – review and editing, resources, project administration. **Peng Liu**: writing – review and editing, supervision. **Xiaodong Li**: writing – review and editing, resources, funding acquisition. **Haitao Wang**: supervision, resources, funding acquisition.

## Conflicts of Interest

The authors declare no conflicts of interest.

## Supporting information




**Supporting file**: advs77871‐sup‐0001‐SuppMat.docx

## Data Availability

The data that support the findings of this study are available from the corresponding author upon reasonable request.
