# Supplementary material for: Pressure‐Activated and Retained Blue Emission in Melamine Through Hydrogen‐Bond and Packing Regulation
Source: Adv Sci (Weinh). 2026 Sep 27:e77871. Online ahead of print. doi: 10.1002/advs.77871 (PMC13616308; doi:10.1002/advs.77871)
Supplement: Supplementary file 1 [file ADVS-9999-e77871-s001.docx]

Supporting Information

Pressure-Activated and Retained Blue Emission in Melamine through Hydrogen-Bond and Packing Regulation

Siyao Wang,^[a][b]^ Weiwei Dong,*^[b]^ Shuo Yang,^[c]^ Jiayue Jiang,^[b]^ Yushuang Wang,^[b]^ Shuailing Ma,*^[c]^ Peng Liu,^[b]^ Xiaodong Li,^[b]^ Haitao Wang*^[a]^

[a]	Siyao Wang, Haitao Wang
School of Materials Science and Engineering, Jilin University, Changchun, 130012, China
E-mail: haitao_wang@jlu.edu.cn

[b]	Siyao Wang, Weiwei Dong, Jiayue Jiang, Yushuang Wang, Peng Liu, Xiaodong Li
High Energy Photon Source, Institute of High Energy Physics, Chinese Academy of Sciences, Beijing, 100049, China
E-mail: dongww@ihep.ac.cn

[c]	Shuo Yang, Shuailing Ma
School of Physical Science and Technology & Institute of High-Pressure Physics, Ningbo University, Ningbo, 315211, China
E-mail: [mashuailing@nbu.edu.cn](mailto:mashuailing@nbu.edu.cn)

Table of Contents

[Section A: Materials and Experimental Methods	2](#_Toc236298478)

[Section B: Ambient Characterization and Pressure-Dependent Optical Properties	4](#_Toc236298479)

[Section C: High-Pressure Vibrational Spectroscopic Studies	6](#_Toc236298480)

[Section D: High-Pressure Powder XRD and Unit-Cell Evolution	9](#_Toc236298481)

[Section E: DFT/TD-DFT Calculations and Hole–Electron Analysis	13](#_Toc236298482)

[Section F: Comparison with reported pressure-treated luminescent materials	20](#_Toc236298483)

[Section G: References	20](#_Toc236298484)

# Section A: Materials and Experimental Methods

**Sample preparation**

Melamine (MA, C3H6N6) was purchased from Innochem with a purity of 99%. The ambient crystal structure was obtained from the Cambridge Crystallographic Data Centre (CCDC No. 1210950) for structural reference. High pressure was generated using a symmetric diamond anvil cell equipped with 400 μm culet diamonds. A T301 stainless-steel gasket was pre-indented to approximately 45 μm, and a hole of 170 μm was drilled as the sample chamber. A small ruby sphere was loaded with the sample for pressure calibration.^[50]^ Silicone oil was used as the pressure-transmitting medium for PL, UV–vis absorption, Raman, and synchrotron XRD measurements, and KBr was used for FTIR measurements.

***In situ* high-pressure measurements**

All high-pressure measurements were carried out at room temperature. For pressure-dependent PL measurements, the excitation power, integration time, optical path, and collection geometry were kept as constant as possible to allow reliable comparison of relative intensities. PL spectra were collected with an Ocean Optics QE65000 spectrometer under 365 nm excitation, and optical photographs were recorded under identical exposure conditions. Time-resolved PL decay curves were measured with a FLIM300 system equipped with a 375 nm pulsed diode laser. UV–vis absorption spectra were recorded using an Ocean Optics QE Pro spectrometer coupled to a deuterium-halogen light source.

High-pressure FTIR spectra were collected using the Fourier Transform Infrared Spectrometer (FTIR Spectrometer, Bruker optics), using a LN-MCT detector and a KBr beamsplitter which can obtain the signals of mid-infrared region within 600-5000 cm^-1^. Raman spectra were collected with a Mono Vista CRS3 Raman spectrometer using a 785 nm excitation laser, with the laser power kept sufficiently low to avoid sample damage.

In situ high-pressure synchrotron XRD measurements were carried out at beamline ID31 of the High Energy Photon Source (Beijing, China) with an incident X-ray wavelength of 0.4133 Å and beam focus of 3 × 2 μm^2^ (horizontal × vertical) at full width at half maximum (FWHM). The XRD patterns were collected using Eiger2 XE CdTe 16M detector. Two-dimensional diffraction images were calibrated with a standard sample and integrated into one-dimensional profiles using Dioptas.^[51]^ Lattice parameters and unit-cell volumes were extracted by Le Bail profile fitting using GSAS-II.^[52]^

**Data analysis and calculations**

**PL decay analysis**. The time-resolved PL decay curves were fitted using a multi-exponential decay function:

$$I(t)=\sum A_{i}\exp(-t/\tau_{i})$$

where $A_{i}$and$\tau_{i}$ represent the amplitude and lifetime of each decay component, respectively. The average lifetime was calculated according to:

$$\tau_{avg}=\frac{(\sum A_{i}\tau_{i}^{2})}{(\sum A_{i}\tau_{i})}$$

The same fitting model was used for all pressure points to ensure reliable comparison of the pressure-dependent lifetime evolution.

**Optical band-gap analysis**. The pressure-dependent optical band gaps of MA were estimated from the UV–vis absorption spectra using Tauc plots. Assuming a direct allowed transition, *E_g_* was obtained according to:

where *α* is the absorption coefficient, *hν* is the photon energy, *A* is a proportionality constant, and *E_g_* is the optical band gap. The linear region of the Tauc plot was extrapolated to the energy axis to determine *E_g_*.

**Le Bail profile analysis.** The one-dimensional synchrotron PXRD patterns were analyzed by Le Bail profile fitting using GSAS-II. The scale factor, background coefficients, crystallite-size parameter, and unit-cell parameters *a*, *b*, *c*, and *β* were refined. The fitting range was approximately 3.206–13.000° in 2θ, with the background described by a ten-term Chebyshev-1 function. The zero shift was fixed at the value determined from the CeO₂ calibration, while the sample-displacement and instrumental-profile parameters were kept fixed. No atomic coordinates, occupancies, or atomic displacement parameters were refined. The experimentally determined ambient-pressure monoclinic *P*2₁/*a* cell of the same sample was used as the initial indexing model, and the converged lattice parameters at each pressure point were used as the starting values for the subsequent point. Quantitative Le Bail analysis was restricted to pressures below 20 GPa, where the crystalline reflections remained sufficiently resolved. Above 20 GPa, pronounced peak broadening and diffuse scattering indicate substantial loss of long-range crystalline order and partial amorphous character; therefore, unit-cell parameters are not reported in this pressure range.

**Theoretical calculations**. DFT and TD-DFT calculations were performed using Gaussian 16 at the B3LYP/6-31G(d) level. The MA monomer, π-stacked dimer, and hydrogen-bonded aggregates were extracted from the crystal structure to evaluate the role of different local molecular environments. Excitation energies, oscillator strengths, and transition configurations were analyzed. Natural transition orbitals and hole–electron distributions were further processed using Multiwfn.

**Hirshfeld surface analysis**. Hirshfeld surface and two-dimensional fingerprint analyses were performed using CrystalExplorer to evaluate the intermolecular contacts in the MA crystal.

# Section B: Ambient Characterization and Pressure-Dependent Optical Properties

**Figure S1.** Ambient structural and optical characterization of MA. (a) Observed and calculated profiles, difference curve, and Bragg reflection positions obtained from Le Bail profile fitting of the laboratory Cu Kα PXRD pattern. The fitted lattice parameters are a=10.5956(20) Å, b=7.47635(47) Å, c=7.2808(12) Å, β=112.1635(32)°, and V=533.992(81) Å³. (b) UV–vis absorption spectrum of MA at ambient conditions.

**Figure S2.** Reproducibility, temporal evolution, and thermal response of the pressure-released PL state of MA. (a) Pressure-dependent PL intensity and emission maximum obtained from three independently loaded samples during compression and decompression, showing reproducible enhancement–attenuation–retention behavior. (b) PL spectra of the recovered MA measured on days 0, 1, 2, 3, 4, 6, and 7 under identical measurement conditions. (c) Corresponding PL enhancement factor relative to pristine MA as a function of storage time. (d) Room-temperature PL spectra of pristine MA and the recovered sample before and after thermal treatment at 55 and 100 °C.

**Figure S3.** Pressure-dependent absorption spectra and optical images of MA. UV–vis absorption spectra of MA during compression (a) and decompression (b). (c) Optical images of MA at selected pressure.

**Figure S4**. Time-resolved PL decay behavior of MA during decompression. Time-resolved PL decay curves (a) and average lifetime evolution (b).

# Section C: High-Pressure Vibrational Spectroscopic Studies

**Figure S5.** Raman spectra of MA during compression.

**Figure S6**. Raman spectra of MA during decompression.

**Figure S7.** Comparison of low-frequency Raman spectra of MA before and after pressure treatment.

**Figure S8.** Low-pressure evolution of the low-frequency Raman modes of MA during compression. (a) Low-frequency Raman spectra collected at 1 atm and 1.1, 2.0, 3.8, and 5.6 GPa. (b) Pressure-dependent Raman shifts of the reliably tracked low-frequency modes. The solid lines are guides to the eye.

**Figure S9.** Pseudo-Voigt profile fitting of the low-frequency Raman spectra of MA at (a) 1 atm, (b) 1.1 GPa, (c) 2.0 GPa, (d) 3.8 GPa, (e) 5.6 GPa.

**Figure S10**. FTIR spectra of MA during compression (a) and decompression (b) processes.

**Figure S11**. (a) FTIR spectra in the 1400–1800 cm⁻¹ region and FTIR spectra in the N–H stretching region of 3300–3500 cm⁻¹. (b) Pressure-dependent wavenumber evolution of N–H-related vibrational modes in MA.

# Section D: High-Pressure Powder XRD and Unit-Cell Evolution

**Figure S12**. *In situ* high-pressure powder XRD patterns of MA.

**Figure S13.** Le Bail profile fitting of the pressure-dependent synchrotron PXRD patterns of MA.

**Figure S14**. Comparison of the initial and recovered lattice states of MA. (a) Normalized synchrotron PXRD patterns collected before compression and after pressure release. (b) Overlay of the initial and recovered monoclinic unit cells viewed along the b axis, together with the relative changes in the lattice parameters and unit-cell volume.

**Figure S15**. XRD line-shape analysis of selected diffraction components of MA before and after pressure treatment. Pseudo-Voigt profile fitting of the selected components Q1, Q2, and Q3 in the (a–c) initial and (d–f) recovered states.

**Figure S16**. Relative increase in the fitted FWHMs of the selected diffraction components after decompression. The initial and recovered profiles were analyzed using the same fitting conditions.

**Table S1**. Pressure-dependent unit-cell parameters of MA derived from powder XRD pattern analysis.

| Pressure (GPa) | Indexing Model | a (Å) | b (Å) | c (Å) | β (°) | V (Å^3^) |
| --- | --- | --- | --- | --- | --- | --- |
| 1 atm | P2_1_/a | 10.5772(27) | 7.49264(59) | 7.2740(15) | 112.2316(46) | 533.615(46) |
| 1.6 | P2_1_/a | 10.3227(40) | 7.43835(68) | 7.0770(24) | 114.5457(68) | 494.293(63) |
| 3.5 | P2_1_/a | 10.1015(86) | 7.38382(77) | 6.9095(37) | 116.585(11) | 460.878(76) |
| 5.2 | P2_1_/a | 9.858(32) | 7.3120(25) | 6.797(15) | 117.724(42) | 433.70(21) |
| 7.4 | P2_1_/a | 9.852(15) | 7.28439(97) | 6.7193(76) | 117.766(21) | 426.707(97) |
| 9.1 | P2_1_/a | 9.767(30) | 7.2383(28) | 6.648(14) | 118.311(39) | 413.78(20) |
| 10.5 | P2_1_/a | 9.690(34) | 7.2643(26) | 6.629(16) | 118.793(44) | 408.96(20) |
| 12.0 | P2_1_/a | 9.566(30) | 7.2066(41) | 6.586(16) | 119.065(41) | 396.85(26) |
| 13.5 | P2_1_/a | 9.432(29) | 7.3180(32) | 6.567(15) | 118.702(40) | 397.58(20) |
| 15.2 | P2_1_/a | 9.218(25) | 7.1857(10) | 6.574(13) | 120.192(33) | 376.42(13) |
| 16.9 | P2_1_/a | 9.134(30) | 7.1365(22) | 6.541(17) | 119.737(41) | 370.22(17) |
| 19.0 | P2_1_/a | 9.221(43) | 7.1424(18) | 6.480(23) | 120.596(57) | 367.36(17) |
| R-1 atm | P2_1_/a | 10.465(14) | 7.3706(13) | 7.0856(74) | 113.484(22) | 501.28(18) |

# Section E: DFT/TD-DFT Calculations and Hole–Electron Analysis

**Figure S17.** Calculated excitation energies, oscillator strengths, and S₁ NTOs of the reference MA models. (a) and (b) represent selected S₁ NTO pairs of the monomer and π–π dimer models, respectively. (c) and (d) are calculated excitation energies and oscillator strengths of the monomer and π–π dimer models, respectively.

**Figure S18.** S₁ hole, electron, and transition-density distributions of the (a) monomer and (b) π–π dimer models.

**Figure S19**. Representative S₁ NTO pairs of the hydrogen-bonded aggregate models with hydrogen-bond shortening of (a) 0.00 Å, (b) 0.05 Å, (c) 0.10 Å, and (d) 0.15 Å. The values beside the arrows indicate the corresponding NTO contributions.

**Figure S20**. Calculated excitation energies and oscillator strengths of the hydrogen-bonded aggregate models with hydrogen-bond shortening of (a) 0.00 Å, (b) 0.05 Å, (c) 0.10 Å, and (d) 0.15 Å respectively.

**Figure S21**. S₁ hole, electron, and transition-density distributions of the hydrogen-bonded aggregate models with hydrogen-bond shortening of (a) 0.00 Å, (b) 0.05 Å, (c) 0.10 Å, and (d) 0.15 Å.

**Table S2.** Calculated excitation parameters and NTO compositions of MA models.

| Hydrogen-bond shortening (Å) | Model | State | Excitation energy (eV) | Wavelength (nm) | Oscillator strength | NTO compositions |
| --- | --- | --- | --- | --- | --- | --- |
| 0.00 | monomer | S_1_ | 5.6648 | 218.87 | 0.004 | NTO33→NTO34(0.595)/NTO32→NTO35(0.405) |
|  | π-π dimer | S_1_ | 5.6497 | 219.45 | 0 | NTO66→NTO67(0.395)/NTO65→NTO68(0.265)/NTO64→NTO69(0.224) |
|  | H-bonded aggregate | S_1_ | 5.4463 | 227.65 | 0.0093 | NTO99→NTO100(0.857) |
| 0.05 | H-bonded aggregate | S_1_ | 5.4231 | 228.62 | 0.0096 | NTO99→NTO100(0.870) |
| 0.10 | H-bonded aggregate | S_1_ | 5.3972 | 229.72 | 0.0098 | NTO99→NTO100(0.882) |
| 0.15 | H-bonded aggregate | S_1_ | 5.3681 | 230.97 | 0.0099 | NTO99→NTO100(0.894) |

**Table S3.** Hole–electron analysis parameters of MA models.

| Hydrogen-bond shortening (Å) | Model | State | S_m^[a]^ | S_r^[b]^ | D (Å) ^[c]^ | t (Å) ^[d]^ | HDI^[e]^ | EDI^[f]^ |
| --- | --- | --- | --- | --- | --- | --- | --- | --- |
| 0.00 | monomer | S_1_ | 0.5296 | 0.8089 | 0.211 | -1.113 | 10.48 | 9.78 |
|  | π-π dimer | S_1_ | 0.5435 | 0.8227 | 0.016 | -2.03 | 7.39 | 6.84 |
|  | H-bonded aggregate | S_1_ | 0.3819 | 0.6594 | 1.27 | -0.186 | 12.45 | 10.67 |
| 0.05 | H-bonded aggregate | S_1_ | 0.3718 | 0.64675 | 1.320 | -0.142 | 12.52 | 10.73 |
| 0.10 | H-bonded aggregate | S_1_ | 0.3612 | 0.63324 | 1.377 | -0.093 | 12.58 | 10.79 |
| 0.15 | H-bonded aggregate | S_1_ | 0.3480 | 0.61711 | 1.449 | -0.022 | 12.73 | 10.84 |

[a] S_m denotes the integral overlap index between hole and electron distributions. [b] S_r represents the spatial overlap degree between hole and electron distributions. [c] D is the centroid distance between hole and electron distributions. [d] The t index evaluates the charge-separation tendency of an excited state; larger positive values indicate more pronounced charge separation, whereas values close to or below zero suggest a more locally redistributed excitation. [e] HDI and [f] EDI describe the spatial delocalization extents of the hole and electron distributions, respectively.

# Section F: Comparison with reported pressure-treated luminescent materials

**Table S4.** Comparison of representative pressure-treated luminescent materials exhibiting retained emission after decompression.

| Material | Retained emission after decompression | Proposed origin | Material characteristics | Ref. |
| --- | --- | --- | --- | --- |
| TPA | Retained enhanced blue emission | Strengthened hydrogen bonding and slipped π–π packing | Simple aromatic small molecule | [25] |
| IPA | Retained white emission | Retained strengthened hydrogen bonding and modified π-stacking | Simple aromatic small molecule | [26] |
| INA | Retained white and yellow emission after different pressure treatments | Hydrogen-bond- and π-stacking-regulated intermolecular charge transfer | Simple heteroaromatic small molecule | [27] |
| BA | 9-fold retained blue-white PL enhancement | Hydrogen-bond-network reconfiguration and pressure-induced structural disorder | Nonconventional organic luminophore | [28] |
| Zn-IPA | 16-fold retained blue or white PL enhancement | Strengthened C–H···π interactions and metal–ligand coordination reconstruction | Metal–organic framework | [29] |
| MA, this work | 35-fold retained enhancement in integrated blue PL intensity | Pressure-modified N–H···N hydrogen-bonded packing and vibrational environment retained after decompression | Commercially available, non-conjugated molecular crystal | This work |

The reported enhancement factors were obtained under different experimental conditions and are listed only as literature-reported values; they should not be regarded as directly comparable quantitative performance metrics.

# Section G: References

[50] H. K. Mao, J. Xu, P. M. Bell, “Calibration of the ruby pressure gauge to 800 kbar under quasi-hydrostatic conditions” *Journal of Geophysical Research: Solid Earth* **91**, (1986): 4673–4676.
[51] C. Prescher, V. B. Prakapenka, “DIOPTAS: a program for reduction of two-dimensional X-ray diffraction data and data exploration” *High Pressure Research* **35**, (2015): 223–230.
[52] B. H. Toby, R. B. Von Dreele, “GSAS-II: the genesis of a modern open-source all purpose crystallography software package” *Journal of Applied Crystallography* **46**, (2013): 544–549.
